# Biomarkers of the Response to Immune Checkpoint Inhibitors in Metastatic Urothelial Carcinoma

**DOI:** 10.3389/fimmu.2020.01900

**Published:** 2020-08-25

**Authors:** Siteng Chen, Ning Zhang, Tao Wang, Encheng Zhang, Xiang Wang, Junhua Zheng

**Affiliations:** ^1^Department of Urology, Shanghai General Hospital, Shanghai Jiao Tong University School of Medicine, Shanghai, China; ^2^Department of Urology, Ruijin Hospital, Shanghai Jiao Tong University School of Medicine, Shanghai, China

**Keywords:** metastatic urothelial carcinoma, *PD-L1*, response, machine learning, nomogram

## Abstract

The mechanisms underlying the resistance to immune checkpoint inhibitors (ICIs) therapy in metastatic urothelial carcinoma (mUC) patients are not clear. It is of great significance to discern mUC patients who could benefit from ICI therapy in clinical practice. In this study, we performed machine learning method and selected 10 prognostic genes for constructing the immunotherapy response nomogram for mUC patients. The calibration plot suggested that the nomogram had an optimal agreement with actual observations when predicting the 1- and 1.5-year survival probabilities. The prognostic nomogram had a favorable discrimination of overall survival of mUC patients, with area under the curve values of 0.815, 0.752, and 0.805 for ICI response (ICIR) prediction in the training cohort, testing cohort, and combined cohort, respectively. A further decision curve analysis showed that the prognostic nomogram was superior to either mutation burden or neoantigen burden for overall survival prediction when the threshold probability was >0.35. The immune infiltrate analysis indicated that the low ICIR-Score values in mUC patients were significantly related to CD8^+^ T cell infiltration and immune checkpoint-associated signatures. We also identified differentially mutated genes, which could act as driver genes and regulate the response to ICI therapy. In conclusion, we developed and validated an immunotherapy-responsive nomogram for mUC patients, which could be conveniently used for the estimate of ICI response and the prediction of overall survival probability for mUC patients.

## Introduction

Urothelial carcinoma is one of the most aggressive malignancies, with an estimated 81,400 new cases and 17,980 deaths in the United States in 2020 (1). Up to 10–15% of initially diagnosed patients have metastatic urothelial carcinoma (mUC), with a 5-year survival possibility of 5% worldwide (2, 3). Currently, cisplatin-based combination chemotherapy is identified as the standard first-line therapy for mUC patients (4). However, more than 60% of mUC patients are not suitable for cisplatin treatment (5) due to their poor performance status or other comorbidities, including renal dysfunction, hearing loss, and heart failure (6). Hence, there remain tremendous practical demands for the development of optimal treatments for these patients.

Currently, developments in immune checkpoint inhibitor (ICI) therapy targeting programmed cell death 1 (*PD-1*) and *PD-1* ligand (*PD-L1*) have revolutionized the management of mUC (3, 7). Compared with traditional chemotherapies, pembrolizumab, a PD-1 blockade drug, demonstrates robust antitumor activity and improves overall survival (OS) by almost 3 months in patients with advanced urothelial carcinoma (7). Unfortunately, only 20–30% of mUC patients achieve response to ICI therapy, and fewer patients could enjoy durable responses for more than 2 years (8). To date, the mechanisms underlying the resistance to ICI therapy in mUC patients are still not clear, suggesting the urgent clinical need for the identification of predictive biomarkers that could discern mUC patients who could benefit from ICI therapy.

Tumor mutational burden (TMB) (3) and DNA mismatch repair (DDR) (9) have been found to be related to the objective response to ICI therapy in mUC patients. However, limitations remain in the clinical application of TMB and DDR mutation status for mUC patients (10). The prognostic model reported by Guru Sonpavde et al. combined genomic and clinical factors to predict the response to anti-*PD-1*/*PD-L1* therapy among 62 patients with advanced urothelial carcinoma (11), and the results also need further validation due to the limited sample size.

In this study, by performing machine learning and nomogram methods, we aimed to create a nomogram model to predict the ICI response and the OS of mUC patients treated with ICI therapy, which could aid in decision-making in clinical practice.

## Materials and Methods

### Patients and RNA Sequencing Data

IMvigor 210 trial was a clinical study (12) exploring the antitumor activity of the PD-L1 inhibitor atezolizumab in patients with mUC. The clinicopathological and the processed gene expression data of 348 mUC patients in IMvigor210 were retrieved from IMvigor210CoreBiologies, a free data resource based on the R environment (13). The baseline characteristics of the mUC patients included sex, race, and tobacco use history; metastatic sites: lymph node (LN) only, visceral, liver, and others; intravesical therapy (BCG) and chemotherapy (platinum); ICI therapy results: complete response (CR), partial response (PR), stable disease (SD), and progressive disease (PD); OS status; and immunotherapy indicators: PD-L1 expression level in immune cells (ICs), tumor cells (TCs), mutation burden per million base pair (Mb), and neoantigen burden per Mb.

The inclusion criteria were as follows: patients with mUC who were platinum refractory or cisplatin-ineligible and treated with atezolizumab, patients with sufficient therapy results (CR, PR, SD, or PD) and follow-up information, and patients with transcriptome RNA sequencing (RNA-seq) data. Patients with missing information on therapy results or survival data were excluded. Finally, 298 patients who met the abovementioned criteria were included and randomly assigned into a training cohort (200 patients) and a testing cohort (98 patients) for the following analyses.

A total of 134 patients with stage IV bladder cancer were also retrieved from The Cancer Genome Atlas (TCGA), as well as their clinical, RNA-seq, and somatic variant data for verification analysis.

### Prognostic Nomogram Model Establishment

The RNA-seq data were log_2_-transformed before further analysis. Genes with very low expression levels were further filtered out. We used the *limma* package in the R environment to identify differentially expressed genes (DEGs) between ICI response and nonresponse patients with a *p* value < 0.05 and |fold change| >1.5. The ICI response patients were defined as mUC patients with CR or PR results after receiving the *PD-L1* inhibitor atezolizumab, while the patients with SD or PD results were defined as ICI nonresponse patients. The most useful genes for OS prediction were selected from the top 20 upregulated DEGs and the top 20 downregulated DEGs via the least absolute shrinkage and selection operator (LASSO) method (14) in the training cohort using the *glmnet* package in R. A prognostic nomogram model was then established based on the selected predictive genes via the *rms* and *nomogramEx* packages of R in the training cohort.

### Evolution of the Prognostic Nomogram Model

Calibration with bootstrapping was conducted to verify the nomogram-predicted probabilities of 1- and 1.5-year OS by plotting these on the x-axis, with the actual OS plotted on the y-axis. The receiver operating characteristic (ROC) curve was performed to assess the specificity and the sensitivity of the nomogram through the area under the curve (AUC) value. The Kaplan–Meier (KM) curves of OS were compared between the low ICI response score (ICIR-Score) group and the high ICIR-Score group based on the log-rank test. Univariate and multivariate Cox regression analyses were also conducted to determine whether the ICIR-Score was an independent prognostic factor of OS. We also performed decision curve analysis (15) to compare the clinical usefulness of the nomogram, mutation burden (per Mb), and neoantigen burden (per Mb) by quantifying the net benefits at different threshold probabilities though the *rmda* package in R.

### Immune Infiltrates and Potential Mechanism Analysis

We estimated the abundances of 22 types of ICs by using CIBERSORT (16). The infiltration scores of the mUC patients were estimated by an immune cell abundance identifier (17). The immunophenoscore based on the expression of major immunocompetence determinants was directly obtained from The Cancer Immunome Atlas for predicting the clinical benefits of tumor immunotherapy (18).

Immune infiltration-related Gene Ontology (GO) biological process and Kyoto Encyclopedia of Genes and Genomes (KEGG) gene sets were enriched via gene set enrichment analysis (GSEA) (19). We also performed somatic variant analysis to detect differentially mutated genes associated with the ICI response via the *maftools* package (20).

### Statistical Analysis

Continuous variables were compared by a two-tailed Student's *t*-test or a one-way analysis of variance. Pearson's chi-square test was used to analyze categorical variables. Statistical Package for Social Sciences 24.0 software (SPSS Inc., Chicago, IL, USA) and R were used for statistical analysis. A *P*-value < 0.05 was regarded as significant.

## Results

### Clinical Characteristics

The baseline clinical characteristics of the training and the testing cohorts are shown in Table 1. Except for the tumor metastasis sites, there was no significant difference in the other medical traits between the two cohorts, which indicated the good performance of the random assignment of mUC patients to the training and the testing cohorts. The baseline clinical characteristics of the TCGA cohort are shown in Table S1.

**Table 1:** Baseline characteristics of mUC patients in the training cohort and the testing cohort.

**Characteristics**	**All (%)**	**Training cohort (%)**	**Testing cohort (%)**	***p* value**
Sex				0.852
Male	233 (78.2)	157 (78.5)	76 (77.6)	
Female	65 (21.8)	43 (21.5)	22 (22.4)	
Race				0.529
White	270 (90.6)	182 (91.0)	88 (89.8)	
Asian	7 (2.3)	3 (1.5)	4 (4.1)	
Black	9 (3.0)	6 (3.0)	3 (3.1)	
Other	12 (4.0)	9 (4.5)	3 (3.1)	
ICI response				0.438
CR/PR	68 (22.8)	43 (21.5)	25 (25.5)	
SD/PD	230 (77.2)	157 (78.5)	73 (74.5)	
Intravesical BCG administered				0.370
Yes	67 (22.5)	48 (24.0)	19 (19.4)	
No	231 (77.5)	152 (76.0)	79 (80.6)	
Platinum received				0.852
Yes	233 (78.2)	157 (78.5)	76 (77.6)	
No	65 (21.8)	43 (21.5)	22 (22.4)	
Tobacco use				0.642
Yes	200 (67.1)	136 (68.0)	64 (65.3)	
No	98 (32.9)	64 (32.0)	34 (34.7)	
Metastasis sites				0.018
LN only	51 (17.1)	26 (13.0)	25 (25.5)	
Visceral (include liver)	220 (73.8)	157 (78.5)	63 (64.3)	
Other	27 (9.1)	17 (8.5)	10 (10.2)	
ICs level				0.571
IC0	83 (27.9)	54 (27.0)	29 (29.6)	
IC1	113 (37.9)	80 (40.0)	33 (33.7)	
IC2+	102 (34.2)	66 (33.0)	36 (36.7)	
TCs level				0.804
TC0	239 (80.2)	159 (79.5)	80 (81.6)	
TC1	17 (5.7)	11 (5.5)	6 (6.1)	
TC2+	42 (14.1)	30 (15.0)	12 (12.2)	
Mutation burden (per MB)	10.8 ± 9.4	10.4 ± 8.9	11.4 ± 10.3	0.448
Neoantigen burden (per MB)	1.4 ± 1.6	1.4 ± 1.6	1.3 ± 1.7	0.636

*mUC, metastatic urothelial cancer; ICI, immune checkpoint inhibitor; CR, complete response; PR, partial response; SD, stable disease; PD, progressive disease; LN, lymph node; ICs level, PD-L1 expression level on immune cells; TCs level, PD-L1 expression level on tumor cells*.

### Identification of DEGs and Key Prognostic Genes

**Figure 1:**
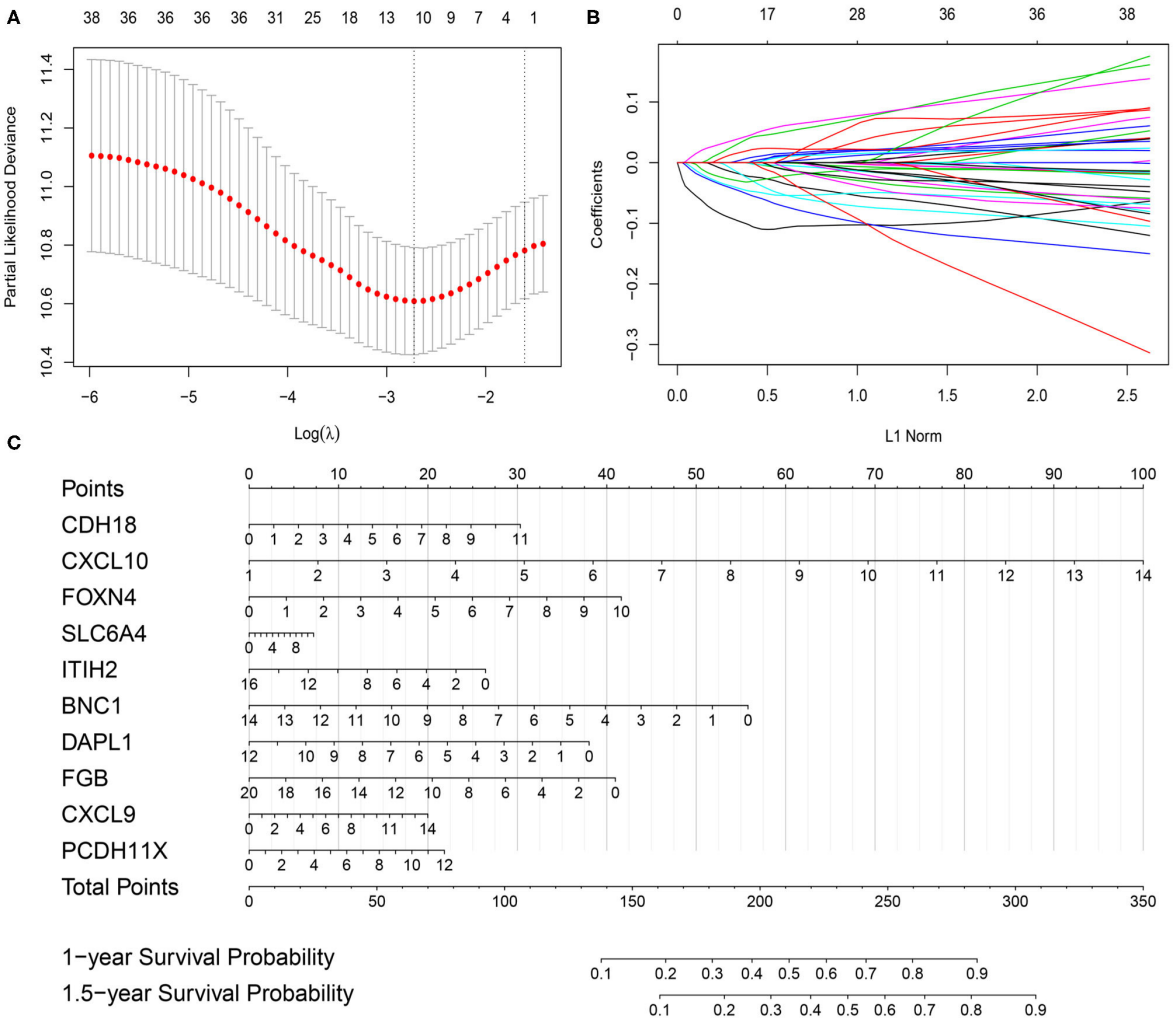
Gene selection via the least absolute shrinkage and selection operator (LASSO) Cox regression model and establishment of the prognostic nomogram model in the training cohort. **(A)** Tenfold cross-validated error. The first vertical line equals the minimum error, whereas the second vertical line shows the cross-validated error within one standard error of the minimum. **(B)** The profile of coefficients in the model at varying levels of penalization plotted against the log (lambda) sequence. **(C)** Nomogram based on selected prognostic genes for the 1- and 1.5-year overall survival predictions in the training cohort. One-year survival probability = 7.9e-08* total points^3^−6.1263e-05 * total points^2^ + 0.010410469 * total points + 0.388686628; 1.5-year survival probability = 1.45e-07 * total points^3^−9.7482e-05 * total points^2^ + 0.016297495 * total points + 0.019957873.

Using the *limma* package in the R environment, we identified 457 DEGs between ICI response (CR and PR) and nonresponse (SD and PD) patients, with a cutoff *p*-value of < 0.05 and |fold change| value over 1.5. Among the 457 DEGs, the top 20 upregulated DEGs and the top 20 downregulated DEGs, which were significantly highly correlated with ICI responsiveness, were further used to identify the most prognostic genes for OS prediction via the LASSO Cox regression method in the training cohort (Table S2). As shown in Figure 1A, the first vertical line pointed at 10, which equaled the minimum 10-fold cross-validated error. After calculating the active coefficients in Figure 1B, 10 key prognostic genes were selected by the LASSO Cox regression model. A further univariate Cox regression analysis was performed and identified that the 10 selected genes were significantly associated with OS in mUC patients receiving ICI therapy (Table 2). Finally, 10 prognostic genes, including six OS-favorable genes (*CDH18, CXCL10, FOXN4, SLC6A4, CXCL9*, and *PCDH11X*) and four OS-detrimental genes (ITIH2, BNC1, DAPL1, and FGB) from the training cohort, were used for further analysis.

**Table 2:** Univariate Cox regression analyses of 10 genes selected via LASSO analysis based on overall survival in the training cohort.

**Characteristics**	**Cox regression analysis HR (95% CI)**	***p* value**	**LASSO coefficient**
**Favorable**			
CDH18	0.887 (0.809–0.973)	0.011	−0.020244481
CXCL10	0.862 (0.807–0.921)	<0.001	−0.098454694
FOXN4	0.877 (0.805–0.956)	0.003	−0.048390155
SLC6A4	0.896 (0.829–0.968)	0.005	−0.031102883
CXCL9	0.882 (0.831–0.936)	<0.001	−0.019969709
PCDH11X	0.859 (0.788–0.938)	0.001	−0.043952722
**Detrimental**			
ITIH2	1.075 (1.012–1.141)	0.018	0.004941391
BNC1	1.052 (1.003–1.103)	0.038	0.03469075
DAPL1	1.098 (1.041–1.158)	0.001	0.041243449
FGB	1.046 (1.002–1.091)	0.040	0.017649583

*LASSO, least absolute shrinkage and selection operator; HR, hazard ratio; CI, confidence interval*.

### Development and Evaluation of the Prognostic Nomogram

The prognostic nomogram for predicting the OS of mUC patients treated with ICI therapy was constructed based on the 10 selected genes in the training cohort. As shown in Figure 1C, each of the 10 selected genes contributed to the total points in the nomogram developed by using the *rms* and the *nomogramEx* packages of R. The total point, which was also called the ICIR-Score, was then acquired by adding the individual points together to predict the 1- and 1.5-year survival probabilities of mUC patients.

**Figure 2:**
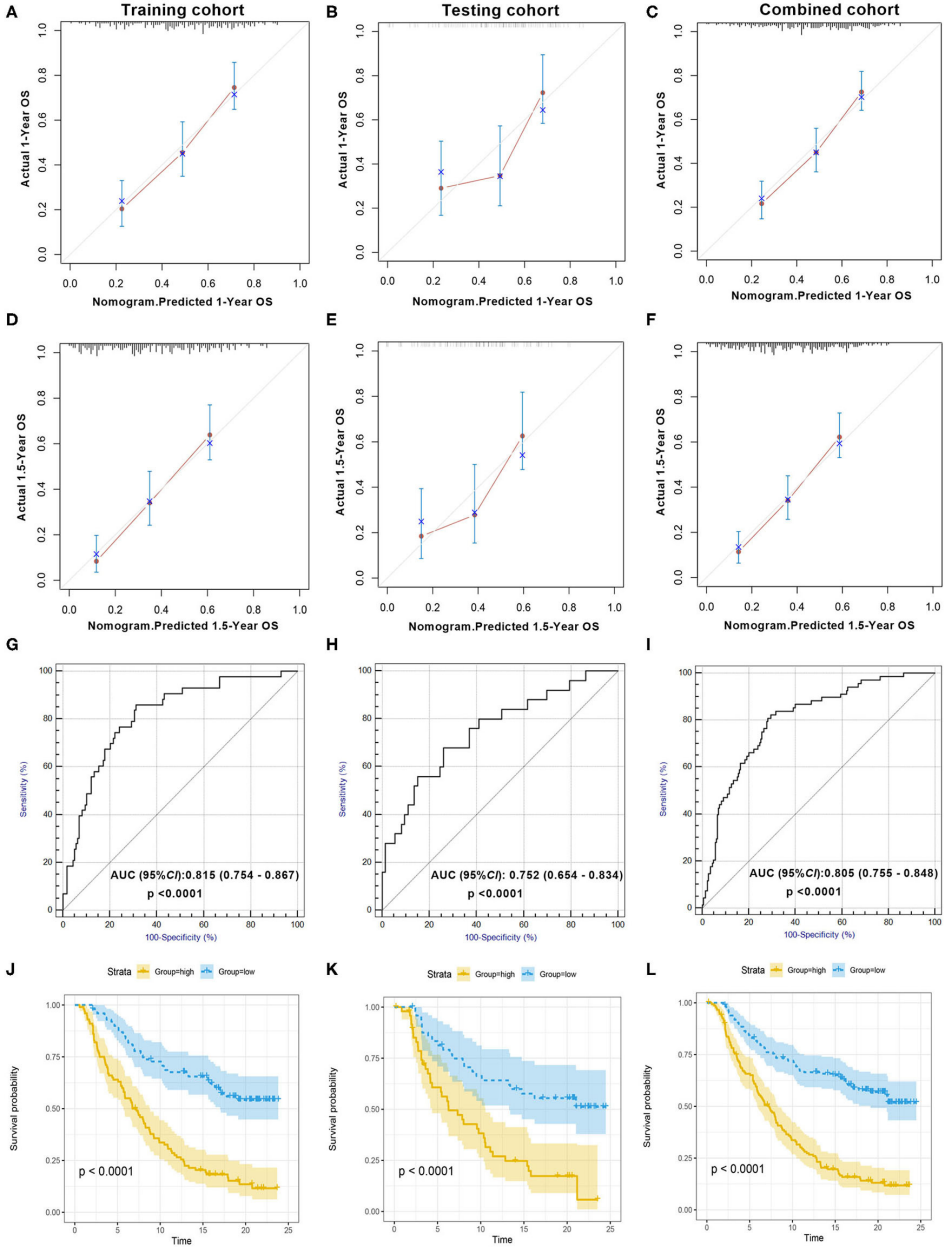
Evaluation of the prognostic nomogram model in the training cohort, testing cohort, and combined cohort. Validity of the predictive performance of the nomogram in the training cohort **(A,D)**, testing cohort **(B,E)**, and combined cohort **(C,F)**. Nomogram-predicted probabilities of 1- and 1.5-year overall survival are plotted on the x-axis; actual overall survival is plotted on the y-axis. ROC curve evaluating the prognostic accuracy of ICI response prediction by nomogram score in the training cohort **(G)**, testing cohort **(H)**, and combined cohort **(I)**. Kaplan–Meier curves of the training cohort **(J)**, testing cohort **(K)**, and combined cohort **(L)**. The median scores of the nomogram in two cohorts were defined as the cutoff value for the high-score and the low-score group, respectively. ROC, receiver operator characteristic; AUC, area under the curve; ICI, immune checkpoint inhibitor.

The calibration plot revealed that the 1- and 1.5-year survival probabilities predicted by our nomogram model had an excellent agreement with the actual observations (Figures 2A,D), indicating that the nomogram had a good ability to accurately predict the survival status of mUC patients treated with ICIs. Less accuracy was found in the testing cohort (Figures 2B,E), which might be due to the small sample size of the testing cohort. The survival probability predicted by the nomogram had an excellent agreement with the actual observations in the combined cohort (Figures 2C,F). A subsequent ROC analysis revealed that our prognostic nomogram had favorable discrimination, with an AUC value of 0.815 (95% confidence interval: 0.754–0.867, *P* < 0.0001) for ICI response prediction in the training cohort (Figure 2G). Validation of the prognostic nomogram was performed in the testing cohort and the combined cohort, with AUC values of 0.752 (95% confidence interval: 0.654–0.834, *P* < 0.0001) and 0.805 (95% confidence interval: 0.755–0.848, *P* < 0.0001), respectively (Figures 2H,I), indicating the good prognostic ability of the nomogram for clinical use.

According to the KM survival analysis, our prognostic nomogram model was able to discriminate patients with poor prognosis from patients with favorable prognosis in both the training and the testing cohorts. The hazard ratios (HRs) of the high- and low-score groups were 3.51 (95% confidence interval: 2.44–5.05, *P* < 0.0001) in the training cohort, 3.11 (95% confidence interval: 1.84–5.26, *P* = 0.002) in the testing cohort, and 3.48 (95% confidence interval: 2.58–4.69, *P* < 0.0001) in the combined cohort (Figures 2J–L). Univariate and multivariate Cox regression analyses in the training cohort indicated that our prognostic ICIR-Score could serve as a predictor of OS in mUC patients, independent of other characteristics (Table 3).

**Table 3:** Univariate and multivariate Cox regression analyses of mUC patients based on nomogram score and other characteristics in the training cohort.

**Characteristics**	**Univariate analysis HR (95% CI)**	***p* value**	**Multivariate analysis HR (95% CI)**	***p* value**
Sex (male vs. female)	1.263 (0.816–1.956)	0.294	/	/
Race (White vs. others)	1.686 (0.824–3.450)	0.153	/	/
Intravesical BCG administered	0.860 (0.565–1.307)	0.480	/	/
Platinum received	0.522 (0.352–0.775)	0.001	0.788 (0.509–1.221)	0.287
Tobacco use history	1.541 (1.041–2.282)	0.031	1.417 (0.935–2.148)	0.100
Metastasis sites				
Visceral vs. LN only	0.585 (0.364–0.940)	0.027	0.597 (0.370–0.963)	0.034
Visceral vs. other	0.796 (0.436–1.453)	0.458	/	/
IC level	1.041 (0.828–1.308)	0.730	/	/
TC level	1.306 (0.818–1.311)	0.772	/	/
Mutation burden (per MB)	1.017 (0.998–1.037)	0.080	/	/
Neoantigen burden (per MB)	1.059 (0.956–1.173)	0.272	/	/
Nomogram score	1.015 (1.011–1.019)	<0.001	1.014 (1.009–1.018)	<0.001

*mUC, metastatic urothelial cancer; LN, lymph node; IC Level, PD-L1 expression on immune cells; TC, PD-L1 expression on tumor cells; HR, hazard ratio; CI, confidence interval*.

**Figure 3:**
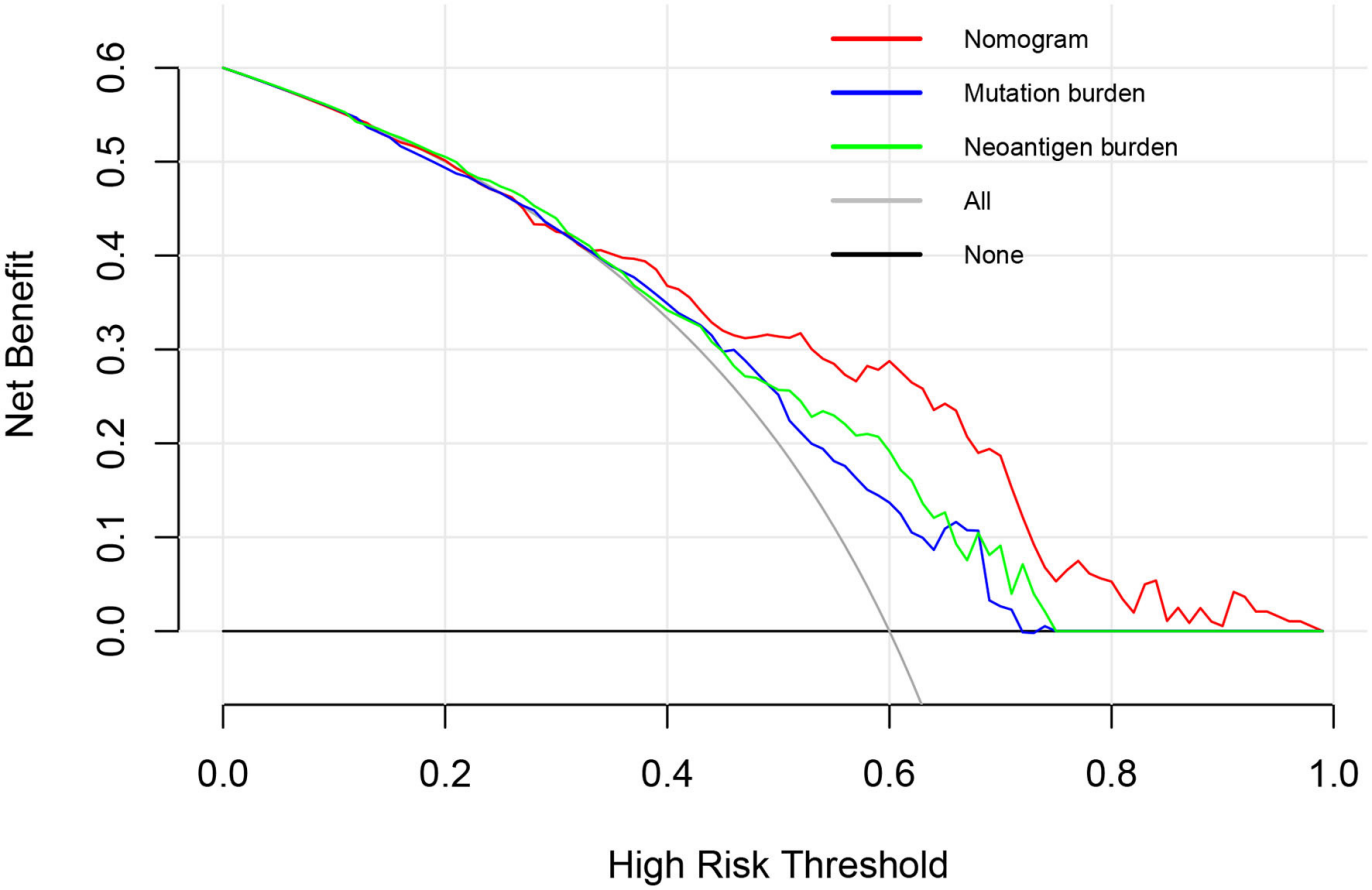
Decision curve analysis comparing overall survival benefits among the nomogram, mutation burden (per MB), and neoantigen burden (per MB). Black line: all victims were dead. Gray line: none of the victims was dead.

A further decision curve analysis showed that, when the threshold probability was >0.35, using the prognostic nomogram for OS prediction showed more benefits than either mutation burden or neoantigen burden (Figure 3).

### Low ICIR-Score Values in mUC Patients Were Associated With CD8^+^ T Cell Infiltration and the Immune-Checkpoint-Associated Signature

**Figure 4:**
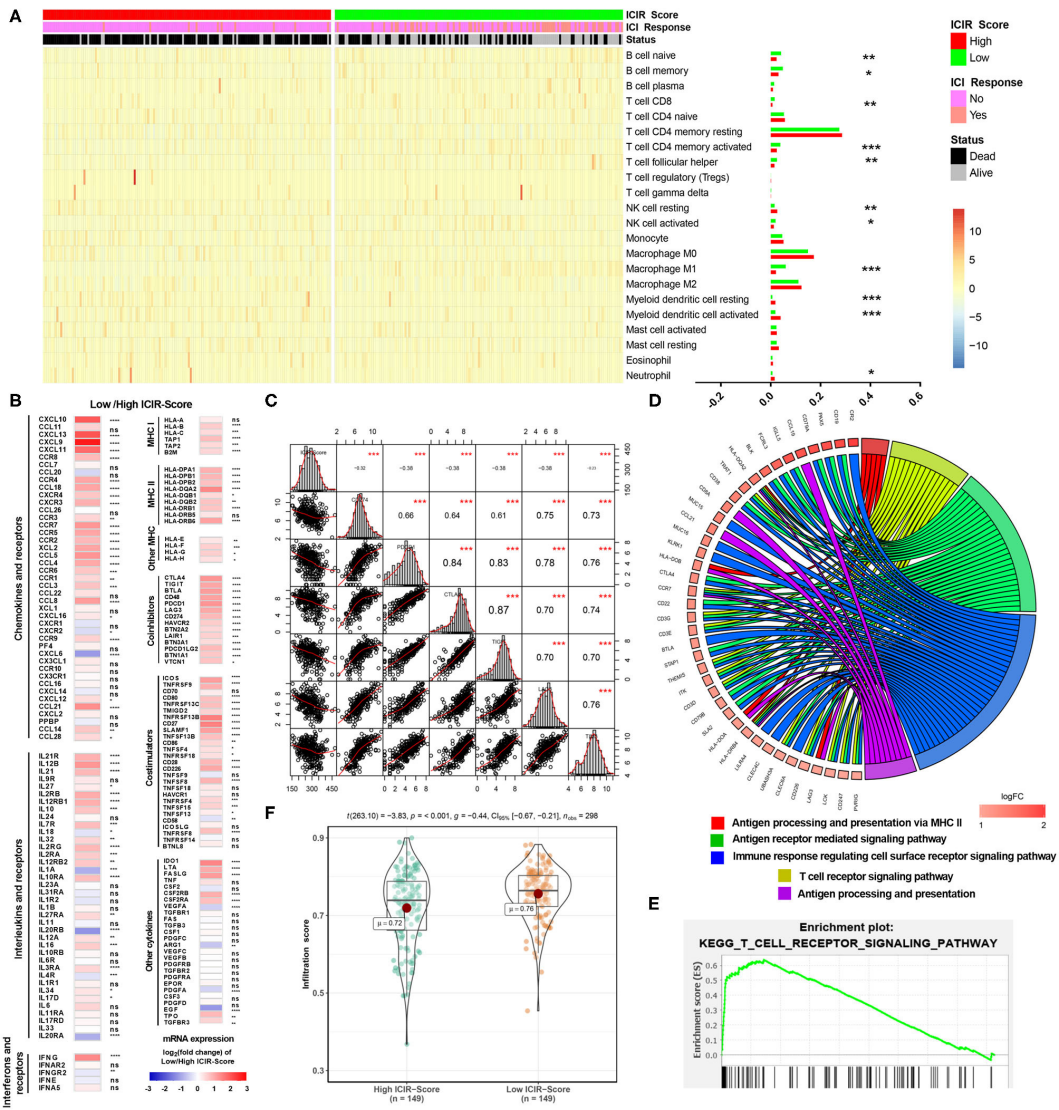
Immune infiltrates and potential mechanism analyses of mUC patients with low ICIR-Score. **(A)** Heatmap and bar graph illustrating the different abundance of 22 immune cell types based on CIBERSORT between mUC patients with high ICIR-Score and low ICIR-Score. **(B)** Expression levels of chemokines, interleukins, interferons, MHC molecules, co-stimulators, co-inhibitors, and other important cytokines and their receptors in mUC patients with low ICIR-Score relative those with high ICIR-Score, shown as log_2_(fold change) of expression value of the respective mRNA. **(C)** Correlation analyses of ICIR-Score and expression levels of immune checkpoint inhibitors in mUC patients. **(D,E)** Gene Ontology and Kyoto Encyclopedia of Genes and Genomes analyses of the differentially expressed genes for mUC patients with low ICIR-Score values using gene set enrichment analysis. **(F)** Comparison of infiltration score of mUC patients between high ICIR-Score group and low ICIR-Score group. mUC, metastatic urothelial cancer; ICIR-Score, immune checkpoint inhibitor response score.

As shown in Figure 4A, different abundances of ICs were identified between mUC patients with low and high ICIR-Score values. For example, mUC patients with low ICIR-Score values had higher abundances of activated CD8^+^ T cells, M1 macrophages, and follicular helper T cells. In addition, the low ICIR-Score values in mUC patients were associated with higher expression levels of some chemokines (Figure 4B), including *CXCL9* and *CXCL10*, which have been proven to attract dendritic cells and CD8^+^ T cells (21). In addition, the higher expression levels of MHC molecules and co-inhibitors were also found to be associated with low ICIR-Score values. Correlation analyses indicated that the mRNA expression levels of ICIs, including CD274 (PD-L1), PDCD1 (*PD-1*), *CTLA-4, LAG-3*, and *TIM-3* (*HAVCR2*), were inversely correlated with the ICIR-Score (Figure 4C).

GSEA revealed that low ICIR-Score values were significantly associated with several immune infiltration-related biological processes, including the T cell receptor signaling pathway, antigen processing and presentation via MHC II, immune response regulating the cell surface receptor signaling pathway, and antigen receptor-mediated signaling pathway (Figures 4D,E). A further analysis revealed that, compared with patients in the high ICIR-Score group, the mUC patients in the low-ICIR-Score group were statistically associated with a higher infiltration score (Figure 4F), which might account for the probable sensitivity to ICI therapy.

**Figure 5:**
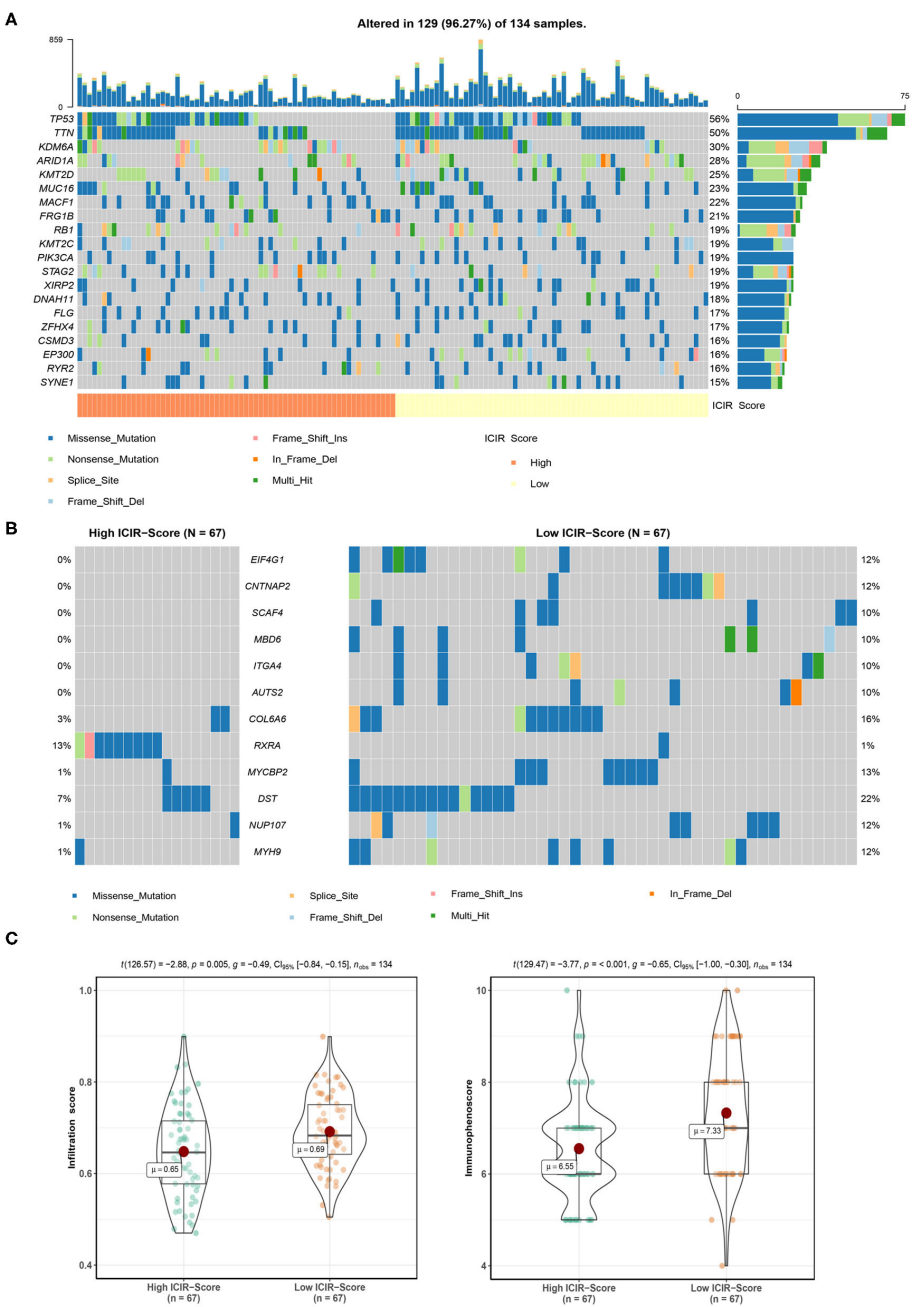
Mutation analysis of mUC patients in The Cancer Genome Atlas cohort. **(A)** Mutation landscape of mUC patients with high ICIR-Score and low ICIR-Score. **(B)** Mutation plot detecting differentially mutated genes between mUC patients in the high ICIR-Score group and the low ICIR-Score group. **(C)** Comparison of infiltration score and immunophenoscore of mUC patients between the high ICIR-Score group and the low ICIR-Score group. mUC, metastatic urothelial cancer; ICIR-Score, immune checkpoint inhibitor response score.

The mutation landscapes of mUC patients with high ICIR-Score values and low ICIR-Score values are shown in Figure 5A. Differentially mutated genes, including *EIF4G1, CNTNAP2, SCAF4, MBD6, ITGA4, AUTS2, COL6A6, MYCBP2, DST, NUP107*, and *MYH9* in the low-ICIR-Score group and *RXRA* in the high-ICIR-Score group (Figure 5B), might act as driver genes and result in differential responses to ICI therapy in mUC. Higher infiltration scores and immunophenoscores were also identified in mUC patients with low ICIR-Score values in the TCGA cohort (Figure 5C).

## Discussion

In this study, we designed and validated a gene signature-based nomogram that was associated with ICI response and could predict the OS of mUC patients treated with ICI therapy. Bic-NaSong et al. identified an immunotherapy-responsive molecular subtype of bladder cancer based on a cluster of 1,627 genes, in which a class 3 cluster was reported to be associated with ICI response because of their high rates of alterations in DDR genes and somatic mutations (22). In this study, our prognostic nomogram comprises only 10 prognostic genes, including six OS-favorable genes and four OS-detrimental genes. Among them, *CDH18, CXCL10, FOXN4, SLC6A4, CXCL9*, and *PCDH11X* are highly associated with ICI response in patients with mUC.

The mUC patients with clinical benefits from ICI therapy were successfully stratified by the prognostic nomogram in both the training cohort and the testing cohort, with HR values of 3.51 and 3.11, respectively, which facilitated the preoperative individualized prediction of ICI response. Accurate predictions of the 1- and 1.5-year survival probabilities of ICI-treated mUC patients were also observed in this study, indicating the good ability of the prognostic nomogram for OS prediction.

ICI response status is a vital clinical factor for patients receiving anti-*PD-1*/*PD-L1* treatment. In a multicenter clinical trial, the objective response rate of cisplatin-ineligible mUC patients treated with pembrolizumab was 24% (23). The objective response rate to another ICI, atezolizumab, was 23%, and the complete response rate was only 9% (3). Our prognostic nomogram performed well in accurately predicting ICI response in mUC patients, with AUC values up to 0.815 in the training cohort, which indicated a good performance for clinical practice.

A further decision curve analysis showed that, when the threshold probability was >0.35, the prognostic nomogram for OS prediction could add more benefit than either mutation burden or neoantigen burden. TMB has been reported to be a significant predictor for the treatment response to ICIs in urothelial cancer in various studies (3, 13, 22). However, high TMB in mUC patients is not sufficient to predict ICI response (9). In addition, whole-exome sequencing for calculating TMB is expensive, which impedes its widespread use in clinical practice (24). Incorporating only 10 genes, our prognostic nomogram seems to be more cost-effective and time-saving in clinical application.

The low ICIR-Score values in mUC patients were found to be associated with CD8^+^ T cell infiltration, conforming to the T cell activation-related biological processes and pathways, which include the T cell receptor signaling pathway and antigen processing and presentation via MHC II.

There are also some notable limitations in our study. First, experimental validation was not performed in this study. The genes used in the nomogram were measured by RNA-seq, which still needs further verification by molecular biology. Second, since this study is a retrospective analysis with data retrieved from IMvigor210CoreBiologies (13), the baseline characteristics of the mUC patients were incomplete. For example, we failed to obtain information about the dose and the schedule of ICIs from IMvigor210CoreBiologies. Third, some potential medical traits, including physical condition and nutritional status, were neglected. Despite these limitations, this study is the largest cohort study of a prognostic nomogram based on gene signatures for ICI efficacy and OS prediction in patients with mUC. Independent of the pathological stage, our prognostic nomogram could help clinicians make more accurate ICI therapy decisions in clinical practice.

In conclusion, based on the 10 prognostic genes associated with ICI therapy, we developed and validated an immunotherapy response nomogram for mUC patients. The prognostic nomogram model has the potential to facilitate the estimation of ICI response and the prediction of OS in patients with mUC, even though experimental validation and prospective validation studies are still needed.

## Data Availability Statement

Publicly available datasets were analyzed in this study. This data can be found here: http://research-pub.gene.com/IMvigor210CoreBiologies/.

## Ethics Statement

Ethical review and approval was not required for the study on human participants in accordance with the local legislation and institutional requirements. Written informed consent for participation was not required for this study in accordance with the national legislation and the institutional requirements.

## Author Contributions

XW, JZ, and SC conceived and designed the study. EZ and TW performed the data collection. SC and NZ analyzed the data and wrote the paper. JZ and SC revised the paper. All the authors read and approved the final manuscript.

## Conflict of Interest

The authors declare that the research was conducted in the absence of any commercial or financial relationships that could be construed as a potential conflict of interest.
