# Supplementary material for: Biomarkers of the Response to Immune Checkpoint Inhibitors in Metastatic Urothelial Carcinoma
Source: Front Immunol. 2020 Aug 25;11:1900. doi: 10.3389/fimmu.2020.01900 (PMC7477044; doi:10.3389/fimmu.2020.01900)
Supplement: Supplementary file 1 [file Table_1.DOCX]

**Table S1.** Demographic, clinical and pathological information of the TCGA cohort.

|  | **TCGA Cohort** |
| --- | --- |
| **No. of patients** | 134 |
| **Age(year)** |  |
| Mean | 70.2 |
| Range | 44.2～90.1 |
| **Gender** |  |
| Male | 99(73.9%) |
| Female  **Race**  White  Black  Asian  Unknow | 35(26.1%)  121(90.3%)  7(5.2%)  2(1.5%)  4(3.0%) |
| **Stage** |  |
| Ⅳ | 134(100%) |
| **Vital status** |  |
| Living | 48(35.8%) |
| Deceased | 86(64.2%) |

TCGA, the Cancer Genome Atlas.

**Table S2** Top differentially expressed genes between immunocheckpoint inhibitor therapy response and non-response patients

| **Gene** | **Log_2_FC** | **AveExpr** | **t** | **P.Value** | **adj.P.Val** | **B** |
| --- | --- | --- | --- | --- | --- | --- |
| **Upregulated** |  |  |  |  |  |  |
| PAX6 | 1.401055 | 5.175908 | 3.207459 | 0.001484 | 0.103381 | -1.18797 |
| CXCL9 | 1.392588 | 8.055148 | 3.774403 | 0.000193 | 0.073402 | 0.539985 |
| GABRA3 | 1.356533 | 6.265849 | 3.617833 | 0.000348 | 0.073402 | 0.037211 |
| KRT20 | 1.35291 | 8.167143 | 2.206737 | 0.028089 | 0.29149 | -3.59263 |
| TAC3 | 1.27015 | 5.752414 | 2.661139 | 0.008207 | 0.184384 | -2.60532 |
| PAX3 | 1.243463 | 2.003551 | 3.382354 | 0.000814 | 0.087537 | -0.68237 |
| CDH18 | 1.210452 | 1.711322 | 3.49202 | 0.000551 | 0.07883 | -0.35276 |
| CDKN2A | 1.196805 | 6.022868 | 3.080141 | 0.002261 | 0.116752 | -1.54035 |
| CXCL10 | 1.176332 | 8.516647 | 3.614866 | 0.000352 | 0.073402 | 0.027873 |
| MAGEA3 | 1.171768 | 5.1236 | 2.029658 | 0.043273 | 0.339312 | -3.92925 |
| FSTL5 | 1.153793 | 2.649801 | 3.360213 | 0.00088 | 0.087537 | -0.74774 |
| FOXN4 | 1.135586 | 1.776119 | 3.694154 | 0.000262 | 0.073402 | 0.279887 |
| PCDH11X | 1.119182 | 4.655168 | 3.700609 | 0.000256 | 0.073402 | 0.30062 |
| FUT9 | 1.111118 | 5.148091 | 2.884042 | 0.00421 | 0.143486 | -2.05699 |
| YBX2 | 1.04397 | 3.607798 | 3.551755 | 0.000444 | 0.078418 | -0.16917 |
| LIN28B | 1.036082 | 2.230933 | 2.801583 | 0.005416 | 0.157691 | -2.26469 |
| SLC6A4 | 1.031443 | 4.371872 | 3.306978 | 0.001058 | 0.091074 | -0.90331 |
| IFNG | 1.030657 | 2.177332 | 4.305753 | 2.26E-05 | 0.041771 | 2.38748 |
| CSAG1 | 1.028297 | 4.167374 | 2.054028 | 0.040839 | 0.33268 | -3.88454 |
| KIF1A | 1.026785 | 4.548268 | 2.442891 | 0.015147 | 0.236732 | -3.10155 |
| **Downregulated** | |  |  |  |  |  |
| HP | -1.10429 | 5.649823 | -2.32017 | 0.021002 | 0.264317 | -3.36273 |
| UGT1A7 | -1.10653 | 8.663551 | -2.3134 | 0.021376 | 0.2658 | -3.37676 |
| FAT2 | -1.14699 | 10.99856 | -2.72053 | 0.006898 | 0.174981 | -2.4633 |
| ITIH2 | -1.18023 | 2.735609 | -3.14354 | 0.001836 | 0.110957 | -1.36654 |
| ANXA10 | -1.18489 | 6.815983 | -2.07568 | 0.038775 | 0.325238 | -3.84438 |
| ALDOB | -1.19872 | 2.228224 | -2.95191 | 0.003407 | 0.137047 | -1.8818 |
| MMP13 | -1.20149 | 8.439428 | -2.61457 | 0.009385 | 0.196206 | -2.7146 |
| FGA | -1.22301 | 2.494836 | -2.62998 | 0.008979 | 0.192241 | -2.67864 |
| BNC1 | -1.25103 | 4.640017 | -2.77929 | 0.005791 | 0.163282 | -2.31985 |
| HMGA2 | -1.28666 | 5.005382 | -2.99595 | 0.002964 | 0.128898 | -1.76607 |
| HABP2 | -1.29158 | 3.384562 | -2.99965 | 0.002929 | 0.127902 | -1.75625 |
| DAPL1 | -1.3055 | 4.075983 | -3.36443 | 0.000867 | 0.087537 | -0.73532 |
| KRT5 | -1.32887 | 12.51813 | -2.64297 | 0.00865 | 0.188609 | -2.64816 |
| FGB | -1.34826 | 2.828919 | -2.58422 | 0.010232 | 0.202092 | -2.78482 |
| UGT1A9 | -1.38319 | 8.474991 | -2.82628 | 0.005025 | 0.153266 | -2.20307 |
| DSC3 | -1.38978 | 9.70276 | -2.70419 | 0.007238 | 0.177649 | -2.50267 |
| TCN1 | -1.42832 | 7.890266 | -3.00249 | 0.002903 | 0.127354 | -1.74874 |
| S100A2 | -1.44093 | 8.919476 | -3.47506 | 0.000586 | 0.07883 | -0.40436 |
| MMP10 | -1.56582 | 7.338432 | -3.8663 | 0.000135 | 0.073402 | 0.844027 |
| UGT1A10 | -1.58714 | 10.17318 | -3.03901 | 0.002582 | 0.120223 | -1.65136 |
